# Oxidative Stress and Exercise

**DOI:** 10.3390/antiox11050840

**Published:** 2022-04-26

**Authors:** Gareth W. Davison, Conor McClean

**Affiliations:** Sport and Exercise Sciences Research Institute, Ulster University, Newtownabbey BT37 0QB, UK; cm.mcclean@ulster.ac.uk

It is now well-established that regular moderate-intensity exercise training can activate salient cell adaptive properties, leading to a state of oxidative eustress. At the other end of the continuum, both sporadic and high-intensity bouts of exercise can induce an intracellular state of oxidative stress, due primarily to an augmented production of reactive metabolites of oxygen such as the superoxide anion and hydroxyl radical species. Exercise-induced free-radical formation impairs cell function by oxidatively modifying nucleic acids, where DNA damage and insufficient repair may lead to genomic instability. Likewise, lipid and protein damage are significant cellular events that can elicit potentially toxic perturbations in cellular homeostasis.

This Special Issue on *Oxidative Stress and Exercise* has attracted a broad range of 10 articles (three narrative reviews and seven original research manuscripts) with over 11,000 views to date. The original research examined oxidative stress and exercise in the context of ageing, probiotics, metabolic acidosis, antioxidant enzyme capacity, obesity, apoptosis, and genotyping, respectively. Considering ageing, Guerrero and colleagues [1] elucidated the impact of ultramarathon exercise (107.4 km) on oxidative stress (MDA, protein carbonyls) with a particular emphasis on outcomes related to sex and age. Their intriguing data showed that sex and ageing per se are important variables for consideration in ultra-endurance exercise-induced macromolecular damage, with more senior runners (45–53 years) having a much higher level of oxidative stress compared to their junior counterparts (31–37 years). The authors suggest that the difference is perhaps due to a higher percentage of body fat and increased loss of muscle mass in the older athletes. The sex differences observed in this investigation are also notable but require comparative data in similar ultra-endurance events.

The antioxidant capacity of erythrocytes is known to protect against severe oxidative stress, however, despite improved haemodynamic efficiency, the effect of eccentric exercise on erythrocyte antioxidant capacity remains unclear. Using a sedentary human model, Huang et al. [2] utilised cycling exercise to ascertain the difference between concentric versus eccentric exercise on a plethora of biochemical molecules, including O_2_ release capacity, glutathione, glutathione disulfide, glucose and lactate production. Both exercise regimes were shown to increase the metabolic state of glycolysis through glucose and lactate, leading to intracellular acidosis and enhanced O_2_ release from erythrocytes. Moreover, concentric and eccentric exercise increased antioxidant capacity, protecting against severe exercise-evoked circulatory oxidative stress. In keeping with the endogenous antioxidant capacity theme, Martinez-Noguera et al. [3] quantified both oxidised (GSSG) and reduced (GSH) glutathione alongside the well-known enzymatic antioxidant’s catalase and superoxide dismutase in different categories of cyclists, concluding that professional cyclists have a higher catalase and oxidised glutathione capacity, respectively, compared to amateur cyclists. They also provided tentative evidence to suggest that antioxidant status (based on their GSSG/GSH data) may be related to power output.

Efficient electron-donor antioxidants are known to minimise exercise-induced oxidative damage to susceptible macromolecules. In a novel study, Sánchez-Macarro et al., used a probiotic product (*Bifidobacterium longum, Lactobacillus casei*, *Lactobacillus rhamnosus)* as an antioxidant activator to examine high-intensity exercise [4]. Using an impressive array of biomarkers (urinary isoprostane, malondialdehyde, oxidized low-density lipoprotein, urinary 8-hydroxy-2′-deoxiguanosine, protein carbonyls, glutathione peroxidase, superoxide dismutase), the authors determined that the consumption of the three-strain probiotic product for 6 weeks in male cyclists undergoing high-intensity exercise was associated with a reduction in lipid-related oxidative stress biomarkers only, with no change in antioxidant enzyme capacity. They conclude that their findings suggest an antioxidant effect of the probiotic product on underlying oxidative stress mechanisms and their modulation in healthy subjects [4]. Betaine is a natural compound, commercially obtained from sugar beet, and is known to protect mitochondrial and wider cell function. To this end, Yang et al. hypothesized that 2 weeks of betaine supplementation can attenuate apoptosis and exercise-induced oxidative stress [5]. Their study demonstrated that placebo ingestion increases lymphocyte apoptosis immediately following, and 3 h post exhaustive exercise, however, betaine supplementation exhibited no change in either apoptosis or oxidative stress.

Two of the published studies opted to use an animal model to probe the relationship between oxidative stress and exercise in a clinical context. Obesity is known to induce insulin resistance, hyperinsulinemia and trigger oxidative stress. In obese male Wister rats, Gómez-Barroso and colleagues [6] tested the effects of diazoxide (*a K_ATP_ channel activator*) and exercise training, both independently and combined, on muscle contraction and subsequent oxidative stress (ROS were determined by oxidation of the 2′, 7′-dichlorodihydrofluorescein diacetate fluorescent probe). Outcomes showed that exercise and diazoxide both reduce ROS production and lipid peroxidation and improve glutathione redox state. Diazoxide treatment and exercise, independently and combined, also leads to a greater increase in maximum and total tension in the soleus muscle of obese rats, suggesting that the K_ATP_ channels may play a prominent role in muscle function in obesity. Chaudhari et al. [7] determined whether dietary vitamin E and C are viable alternatives to exercise in improving cognitive and motor performance in a mouse model of Alzheimer’s disease risk. Mice expressing human Apolipoprotein E3 (GFAP-ApoE3) or E4 (GFAP-ApoE4) fed an 8-week control or antioxidant-rich diet were subjected to various tests to quantify reflex and motor, cognitive and affective function. The antioxidant diet (and exercise) improved balance, learning, and cognitive flexibility in the GFAP-ApoE3 only group, with the authors concluding that genotyping needs to be considered in interventions designed to improve brain function during ageing, and particularly in neurodegenerative disease.

Three narrative reviews were published in this *Special Issue*. In their review, Zeng et al. [8] summarised the current evidence available for the use of whole dietary strategies to reduce exercise-induced oxidative stress in humans. The review included an overview of 28 studies, with the majority outlining the importance of dietary antioxidant consumption as a scavenging technique to curb exercise-related oxidative stress. Yet, the investigative protocols are heterogeneous in nature, and further enquiry is needed to strengthen the evidence base. Other dietary factors, such as excessive consumption of nutrients, are tightly linked to obesity, where oxidative stress plays a prominent role. For example, nutrients such as glycerol and fatty acids can accumulate in white adipose tissue, while subsequently secreted molecules such as adipocytokines (IL-6, TNF-α) and reactive species can lead to inflammation and oxidative stress. Taherkhani and colleagues propose in their review [9] that antioxidant supplementation can therapeutically help to neutralise oxidative stress by removing reactive oxygen metabolites in animal adipose tissue. The authors acknowledge that data in this domain are currently inconsistent due to differences in study design relating to experimental duration and diversity (strain, age sex) in animal models used [9]. As outlined by McClean and Davison [10], compelling research now documents how the circadian system is essential for the maintenance of homeostasis and glucose metabolism, and disruptions to circadian rhythms may well be instrumental in the development of obesity and other conditions. Although a direct relationship has been established between circadian rhythms and oxidative stress, it appears that the emerging interface between circadian rhythmicity and oxidative stress has not been explored in relation to exercise. In their novel review [10], a summary of the evidence supporting the conceptual link between the circadian clock, oxidative stress/redox homeostasis, and exercise stimuli is offered, while the latter section of the review outlines that carefully designed investigations of this nexus are required to further progress this important area of study.

The guest editors would like to acknowledge all author contributions to the Special Issue *Oxidative Stress in Exercise*. While we commend this Special Issue to the readers of *Antioxidants*, we trust that the work contained within will be viewed as a starting point for further exploration into the complex relationship between oxidative stress and exercise.
